# Understanding the experience of relational accommodation for caregivers of an individual with body dysmorphic disorder: An interpretative phenomenological analysis

**DOI:** 10.1111/papt.70067

**Published:** 2026-04-23

**Authors:** Deanna Fallah, Lucy Hale, Francesca Cicconi, Hannah Frith

**Affiliations:** ^1^ Department of Psychology University of Surrey Guildford UK; ^2^ Present address: Department of Psychology IoPPN, King's College London London UK

**Keywords:** body dysmorphic disorder, caregivers, family accommodation, interpretative phenomenological analysis, lived experience, qualitative research, relational accommodation

## Abstract

**Objectives:**

This study explored experiences of Relational Accommodation (RA) for caregivers and significant others living with an adult with Body Dysmorphic Disorder (BDD) and how they respond to BDD symptoms. BDD is under‐researched. In paediatric and/or obsessive‐compulsive populations, RA has been found to negatively impact the lives of caregivers. To date, very little is understood about RA in caregivers for adults with BDD and how this impacts the phenomenology of BDD. Given the high suicidality rates in BDD populations, and somewhat conservative treatment outcomes, a greater understanding is needed.

**Design:**

Eight caregivers, recruited from a BDD research conference and online support groups, were interviewed online about their experiences cohabiting with a loved one with BDD.

**Methods:**

Interviews were transcribed and subject to Interpretative Phenomenological Analysis (IPA).

**Results:**

Four Group Experiential Themes were interpreted from participants' accounts: RA occurs in the context of Distress; RA and Self‐concept are intertwined; Relational Gains and Losses; and Understanding of RA and BDD changes over time. Participant experiences of RA mirrored the OCD and/or paediatric BDD literature confirming the importance of this experience in BDD and extended existing knowledge by highlighting nuanced differences specific to being a caregiver of an adult with BDD.

**Conclusions:**

Improved parent‐ and clinician‐specific guidance around RA and parent peer support groups should be considered. Future research should seek to recruit a more diverse representation of the adult caregiver experience, including that beyond the parent–child dynamic.

## INTRODUCTION

Body Dysmorphic Disorder (BDD) is a preoccupation with perceived defects in appearance that are not clearly observable to others but cause significant distress and/or impairment in functioning (American Psychiatric Association, 2013). BDD prevalence is difficult to ascertain due to under‐recognition in clinical settings (Hartmann et al., 2017), however is estimated to be between 2% and 3% in the general population. BDD is underrepresented in research, and little attention is given to the relational context of BDD symptoms, particularly in the adult population.

BDD is categorised under ‘Obsessive‐Compulsive and Related Disorders’ (OCRDs) (American Psychiatric Association, 2013; World Health Organization, 1992), reflecting shared clinical features and treatment approaches between BDD and Obsessive‐Compulsive Disorder (OCD). Both are classified by intrusive and obsessive thoughts causing significant distress, accompanied by compulsive behaviours or rituals serving to reduce distress. They share similar sex ratios and high comorbidity rates (Frare et al., 2004). However, their differences have treatment implications; BDD cognitions are specifically appearance related, BDD is thought to cause greater impairment in psychosocial functioning, higher comorbidity with substance use and major depressive disorders, and poorer insight compared to OCD (Didie et al., 2007). Suicidality rates are markedly higher in BDD populations compared to the general population, with individuals four times more likely to experience suicidal ideation and nearly three times more likely to attempt suicide (Angelakis et al., 2016).

The National Institute for Health and Care Excellence (NICE) guidelines for BDD treatment (NICE, 2005) recommend the same psychological treatment as OCD: Cognitive Behavioural Therapy (CBT) with Exposure and Response Prevention (ERP) (American Psychiatric Association, 2013; National Institute for Health and Care Excellence [NICE], 2005). A systematic review and meta‐analysis of randomised controlled trials (RCT) of CBT for adult BDD found that only 48%–54% of sufferers were treatment responders (Harrison et al., 2016), compared to 60%–80% in CBT trials for OCD (Mataix‐Cols et al., 2016; Öst et al., 2015), emphasising the need for improved BDD treatment options. It is important to understand relational factors that influence BDD such as interpersonal dynamics, as this may have important treatment implications.

Relational Accommodation (RA) refers to family members/significant others (SO) adapting their behaviour and responses to their loved one's symptoms, to alleviate their distress (Lebowitz et al., 2016). RA has been used in previous literature as a more inclusive alternative to the term ‘Family Accommodation’ (FA), by recognising the role different relational structures can have in accommodating symptoms, including families without biological/legal connections. RA may perpetuate symptoms by limiting opportunities for individuals to develop distress tolerance (Craske et al., 2008) or challenge their maladaptive beliefs (Storch et al., 2007). A breadth of research explores RA across autism spectrum condition (ASC) (Russell et al., 2013; Storch, Salloum, et al., 2015a; Storch, Zavrou, et al., 2015b) and mental health conditions including anxiety disorders (Lebowitz et al., 2013; Norman et al., 2015; Storch, Salloum, et al., 2015a; Storch, Zavrou, et al., 2015b) and post‐traumatic stress disorder (PTSD) (Fredman et al., 2014, 2016). A primary focus is on OCD, suggesting RA is significantly associated with symptom severity (Hermida‐Barros et al., 2024), functional impairment (Storch et al., 2010) and impacts the accommodators' lives (Stewart et al., 2017). Research largely focuses on parental impact in paediatric OCD. However, Brownings et al. (2023) explored eight young people's experiences of living with a sibling with OCD. Interpretative Phenomenological Analysis (IPA) highlighted accounts of symptom accommodation, frustration, helplessness and distress avoidance, demonstrating that RA can occur in various relational dynamics.

Although RA literature mostly relates to paediatric OCD, research recognises that SO of adults (parents, partners, or non‐family members) accommodate symptoms (Lebowitz et al., 2016). RA behaviours most exhibited by SO of OCD sufferers include participating in compulsions, assisting in trigger avoidance and providing reassurance. RA in co‐habiting parents and spouses is associated with increased stress (Calvocoressi et al., 1995), depression (Benito et al., 2015), a loss of interpersonal relationships (Cooper, 1996), and increased anxiety (Futh et al., 2012). RCTs indicate better treatment outcomes in individuals receiving behavioural therapy compared to only pharmacological intervention for OCD (Romanelli et al., 2014). Preliminary evidence suggests that a multifamily psychological intervention for caregivers of adults with OCD reduces RA through psychoeducation and training, supporting RA‐targeted interventions to reduce OCD symptoms (Maina et al., 2006).

Given the similarities and differences between OCD and BDD and RA's impact in OCD, understanding RA's function and presentation of RA in BDD is important to inform treatment. There are two published qualitative studies exploring RA in BDD. Jassi et al. (2020) interviewed young BDD sufferers, parents and clinicians exploring the types, impact and purpose of RA in BDD. Findings showed similarities to other conditions, such as reassurance seeking and engagement in rituals to reduce distress. Differences included the funding of cosmetic products and procedures, and risk concerns/suicidality as reasons for accommodating symptoms; both likely to be more specific to BDD. These differences warrant further exploration around the nature and impact of RA in BDD. Within the adult BDD population, one study explores the role romantic partners play in cis‐women's appearance‐anxiety, highlighting the perceived unhelpfulness of RA on symptoms (Lumsdale et al., 2024).

To date, little is understood about RA's impact in adult BDD sufferers and co‐habiting loved ones. Considering the high suicidality in BDD populations and somewhat conservative treatment outcomes, better understanding the phenomenology of BDD is needed. Given that multifamily interventions for adult OCD sufferers effectively reduce RA and OCD symptoms, and the diagnostic overlap between OCD and BDD, a better understanding of how RA presents in BDD may inform and potentially improve current BDD treatment options.

This study is the first to explore the experiences of RA for caregivers of adults with BDD. This study aimed to explore: (1) the experiences of family members and SO living with an adult with BDD/appearance‐related anxiety, (2) how family members and SO respond to the symptoms of BDD/appearance‐related anxiety in their relative/loved one.

## METHODS

### Design

This study adopts a qualitative, interpretative design using Interpretative Phenomenological Analysis (IPA). There are three theoretical foundations to this study. (1) Phenomenology, which holds that our lived experience is personal and a function of our relationships to the world/others, and that our emotional experiences are key in sense‐making of the world, particularly when the structure has been disturbed (Smith et al., 2022). This study aimed to establish the core of experiences of living with an adult with BDD and understand individuals' unique perspectives, meanings and relationship to their world. (2) Hermeneutics recognises that participants interpret their own experiences and researchers interpret these accounts within context to uncover deeper meanings (Smith et al., 2022). This process risks researchers' preconceptions overshadowing participants' meaning‐making, underscoring the dynamic, non‐linear nature of IPA and importance of reflexivity. (3) Idiography or a focus on understanding a person's unique meaning of phenomena (Smith et al., 2022), in this case highlighting the uniqueness of their experience of living with an adult with BDD.

### Participants

Sampling was purposive; participants were selected because of their experiences, perspectives and expertise in being a family member/SO of an adult with BDD, and not because they were demographically representative of the larger population (see eligibility criteria in Table 1).

**TABLE 1:** Inclusion and exclusion criteria for participant recruitment.

Inclusion criteria	Exclusion criteria
Participating individuals must be an adult (18+ years)	Individuals who do not have capacity to provide informed consent
Individuals must be a family member/carer/partner/close friend supporting an adult (18+ years) with BDD or appearance‐related anxiety	
Individuals co‐habiting for a minimum of six months with an adult with a primary diagnosis of BDD	
Individuals must have the ability to take part in a 60‐min interview	
Individuals must be English speaking (as this is a doctoral thesis there is no budget for an interpreter)	

Abbreviation: BDD, Body Dysmorphic Disorder.

Only one family member/SO from each family was interviewed. IPA studies generally use small sample sizes, aiming to seek a ‘reasonably homogeneous sample’ for detailed exploration of similarities and differences in perspectives about a topic. In line with recommendations, this study aimed to recruit six to eight participants (Smith et al., 2022). A similar IPA study found eight participants sufficient to provide novel insight into the experiences of siblings of OCD sufferers (Brownings et al., 2023). Participants were recruited via online BDD support groups and at a BDD research conference, using a recruitment poster and an online eligibility screening form. For eligible, consenting participants, the researcher offered an online pre‐interview meeting to familiarise themselves with the researcher, ask questions and check for technical issues. Participants completed a demographics survey.

### Ethics

Ethics approval was granted from the University of Surrey's Research Integrity and Governance Office (RIGO) (EGA ref.: FHMS 22‐23 236 EGA). Participants provided informed consent written prior to, and verbal consent during, the interview. Due to the sensitive nature of the topic, the researcher used a compassionate interview style to minimise potential distress, offered opportunities for interviews to be paused, terminated and/or rescheduled if necessary, and provided a debrief sheet signposting sources of support at the end of the interview. Post‐interview participants received a £20 ‘thank you’ voucher.

### Data collection

Individual, single, in‐depth, semi‐structured interviews were conducted online by researcher DF via Microsoft Teams to facilitate participation by those with caring or accessibility issues. Interviews were up to 60 min, with an additional 30 allocated for breaks. Interviews followed a topic guide co‐produced and piloted with a Service User and Carer advisory group to refine content and wording. Questions were drawn from previous RA studies (Brownings et al., 2023), the Family Accommodation Scale for Obsessive‐Compulsive Disorder Interviewer‐Rated (FAS‐IR; Calvocoressi et al., 1999), and the advisory group. High‐quality IPA data requires balancing guidance with participant‐led exploration. Opening questions elicited participants' understanding of BDD to provide context and shape narratives from their own perspectives (Smith et al., 2022), while later sensitive questions allowed a shift from descriptive to affective responses. Field notes were made during and after the interview with initial reflections, contextual details and non‐verbal expressions.

### Data analysis

Microsoft Teams transcription was utilised during interviews which were later examined alongside audio recordings to correct errors. This research utilised an IPA approach involving detailed analysis of each participant followed by identifying patterns across them, highlighting shared and individual themes (Smith, 1996). IPA was adopted because the research aimed to understand not only the experiences of caregivers/SO of adults with BDD, but also how they made sense of them, including motivations and perceived consequences. Being a lived body in the world was crucial to understanding participants' perspectives, especially expressed emotions indicating what they cared about. The stages of IPA involved: (a) re‐reading the first transcript whilst listening to the audio to identify significant ideas and initial interpretations; (b) analysing these exploratory comments to develop experiential statements and mapping these into Personal Experiential Themes (PETs) and then repeating these initial steps for all transcripts, treating each case individually; (c) identifying similarities and differences across the cases to generate Group Experiential Themes (GETs) which form the basis for a narrative account of participant experiences, highlighting both shared and unique features. There was one data coder (researcher DF). PETs and GETs were refined and revised in consultation with the research team. The SUC advisory group was consulted to refine interpretation and implications of findings. Given the double hermeneutic in IPA, reflexivity is an important mechanism for thoughtful interpretation. The research team brought varied experiences of caregiving to the research, although none had experience of caring for someone with BDD; in addition to this (and varied life experiences and world views) the first author kept a reflexive journal, and reflexive team discussions formed part of the analytic process. This study is reported in line with the Consolidated Criteria for Reporting Qualitative Research (COREQ) guidelines (Tong et al., 2007).

## FINDINGS

This study aimed to explore: (1) The experiences of family members and SO living with an adult with BDD/appearance‐related anxiety, (2) How family members and SO respond to the symptoms of BDD/appearance‐related anxiety in their relative/loved one. Eight participants were recruited and interviewed for between 63 and 100 min (see Table 2 for sample demographics). Although attempts were made to recruit a diverse sample, all participants were White, cis‐female mothers, with an average age of 57. Two romantic partners of an individual with BDD who expressed interest in participating dropped out with no reason provided. One identified as LGBTQIA+ and global majority and another identified as male. Nevertheless, participants shared diverse and detailed accounts of their experiences of living and caring for an adult with BDD, and all showed a detailed understanding of clinical guidelines and relational accommodation, enabling thorough interpretation and reflection.

**TABLE 2:** Participant characteristics.

Participant^a^	Age	Gender	Sexual orientation	Ethnicity	Relationship to person with BDD
Mary	56	Female	Heterosexual	White British	Mother
Kate	61	Female	Heterosexual	White British	Mother
Susan	58	Female	/^b^	White British	Mother
Mae	48	Female	Pansexual	White British	Parent
Amelia	62	Female	Heterosexual	White British	Mother
Robyn	59	Female	/	White British	Mother
Grace	61	Female	Bisexual	White British	Mother
Bailey	57	Female	Heterosexual	White Other	Mother

*Note*: The table summarises demographic characteristics of study participants and their relationship the person with Body Dysmorphic Disorder (BDD).

Abbreviation: BDD, body dysmorphic disorder.

^a^ All participant names are pseudonyms.

^b^ / indicates that the participant preferred not to disclose this information.

**FIGURE 1:**
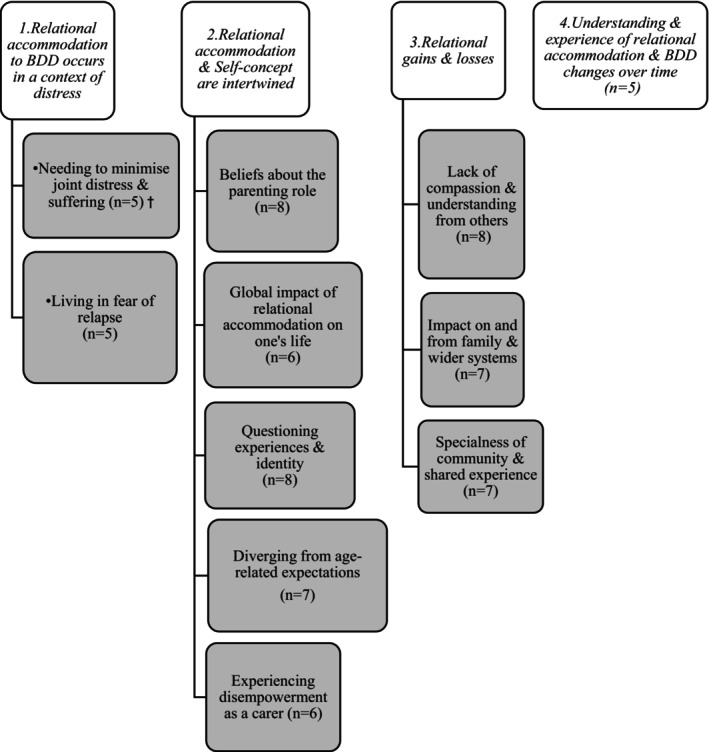
Overview of the four themes and subthemes, which describe carers' experiences of relational accommodation (RA) in the context of Body Dysmorphic Disorder (BDD). ^†^Indicates number of participants contributing to this theme/sub‐theme.

Four GETs were interpreted from participants' accounts: *Response to BDD occurs in a context of Distress, Relational Accommodation and Self‐concept are Intertwined*, *Relational Gains and Losses*, and *Understanding and Experience of Relational Accommodation and BDD changes over time*. Figure 1 provides an overview of the GETs, group‐level subthemes and number of participants who contributed each sub‐theme.

### Group experiential theme 1: ‘Relational accommodation to BDD occurs in the context of distress’

Living with BDD and caring for someone with BDD are both complex, exhausting, and distressing. Participants described how, as mothers, their response to BDD is driven by *needing to minimise joint distress and suffering*. They experience an immediate response to reduce or avoid their child's distress:

I don't know whether in hindsight, buying products for her was the right thing. However, I do know that she was so distressed that it just alleviated her distress. I think that was the motivator at that point was just alleviating her distress because, you know, she would just, she would just be sitting sobbing. Um, and as a parent, you'll just do anything to stop your child being in that state… I think you do, that is what you think because you as a parent, you should be able to make it better for your child really. (Robyn).

Here, RA (buying appearance‐related products) functions to reduce her daughter's distress and to confirm that Robyn is a loving parent. Participants articulated an awareness of the clinical guidance and potential unhelpfulness of RA but reported finding it difficult not to address their child's distress with RA. Susan captured the conflict experienced by participants, contrasting when they are/are not available to support their child: “I'm not on hand so… she has to deal with it [distress] if you know what I mean, whereas if I was there, I would go into the room and say, ‘okay, what can I do to help?'… So maybe (laughs) we don't always, we think we're doing good or whatever, I don't know.” (Susan). Although Susan acknowledged that “we think we're doing good” when engaging in RA, she recognises that this means that her daughter does not have “to deal with it” herself. BDD is a difficult, serious chronic mental health difficulty carrying a high suicide risk, and the distress for the person with BDD and their caregivers should not be under‐estimated as it may be experienced as intolerable by one or both. This was powerfully communicated by Mary, who describes the desperation and loss of hope she felt in the face of her son's distress:

And I wanted him to die. I… if I… I would have killed him. I planned to kill him. I thought of how I could get away with it, very seriously. I'm not saying that light‐heartedly. I'm serious. I would talk to my mum about it in a very rational way, which thinking about it now is crazy. It's crazy to say this, but I used to say to my mum, very seriously, ‘I can, I can do it, I can, I could do it. I could get away with it. I know, what could I do? How could I get away with it?’… ‘He'd be better off dead’. And I used to say, ‘we'd all be better off dead. We'd all be better off if he was dead’. Because, you know, I'd say‐ and I genuinely believed this. I genuinely believed this (Mary).

This extract speaks to Mary's despair in which a violent act is framed as a compassionate response to distress which is depicted as alleviating the suffering of her son, and of the family. Now their situation has become somewhat more manageable over time, she is able to reflect on the gravity of her past intentions.

Like Mary, other participants also described experiencing critical or traumatic moments in relation to their child's BDD‐related distress, including taking them to hospital, police searches, long periods of screaming or crying, and attempted suicides. Even when this point of crisis has passed, carers can feel like they are *living in fear of relapse*. As Mary illustrates:

Say he laughs at something or he drops the remote control onto the floor from his bed or from his desk or whatever. That noise, I think, I am immediately taken back to those days of him screaming. And I'm like… I immediately shout up the stairs, “everything alright?”

This experience of constant hypervigilance around their child's distress, “it's as though I'm waiting for the, for it to start.” (Mary), is also described by participants who report that their child is in ‘remission.’ They fear that their loved one “would not, suddenly not be able to cope.” (Mae) or constantly live with uncertainty that “the rug could be pulled out at any point” and feeling that “we can't really plan anything[…] there's still a possibility that something will go wrong somewhere along the line…” (Robyn). Robyn's long‐lasting response to BDD has been shaped by the unpredictability around the initial onset of BDD and distressing experience of fearing her daughter's relapse.

### Group experiential theme 2: ‘Relational accommodation and self‐concept are intertwined’

For all participants, aspects of RA and their response to their child's BDD influenced how they viewed themselves as carers and their identity more broadly. Additionally, participants recognised that their own *beliefs about the parenting role* in response to BDD, including RA. RA is viewed as an expression of the values they hold around themselves as mothers, including beliefs that sacrificing their own needs and prioritising their child's needs makes them a supportive, compassionate parent. One participant described how RA fits with her understanding of being a good parent, and that she feels judged and misunderstood by clinical guidance which tries to direct her away from this position:

Well it [RA] makes me feel like I'm a better parent because I'm showing empathy and kindness and understanding to my daughter who's poorly… so yeah… oh God, it's emotional (starts tearing up). It is also the role of parents to support and not challenge, ‘cause actually the world is challenging enough and I think I just feel really angry towards the BDD community for not understanding how hard it is for parents’. (Grace)

Here, Grace notes a contradiction between the qualities of being a good parent (who shows empathy, kindness and support) to her vulnerable and unwell daughter, and the requirement to avoid accommodating as advised by the BDD community (like clinicians and other carers). Like this participant, others described feeling “overwhelmed” (Bailey) and having to “constantly question if I'm doing the right thing” (Kate) about the demands on them to avoid RA, when this went against their understanding of what good parenting involves and what they understand their child to need. Furthermore, navigating RA was seen as antithetical to developing and maintaining close relationships with their child, relationships which were seen as indicative of good parenting. Bailey described the “minefield” of navigating “maintaining a connection with him and also, um, help him get better and never really knowing what's the best way to do both”, knowing the two may not co‐exist and feeling concerned things could explode at any point. All participants described a sense of duty as a parent to keep their child safe, and RA being driven by risk concerns. As Robyn observes prioritising RA above clinical guidance, was a way to keep her daughter safe: “she'd have been self‐harming, I'm sure she probably‐ oh, God forbid. So yeah, I think it [RA] did have a positive effect actually thinking about it because I think we kept her safe, I think we, we gave her a reason… to carry on and to not want to harm herself”. For Mae, decision‐making about RA centred both her daughter's and her own safety and avoidance of potentially harmful consequences:

I just had no idea, erm, what to do and I just knew that the (.) lesser evil was to try and provide her safe things than not to provide them and let her kick off (pause). ‘Cause she got very aggressive when she kicked off towards me, towards anything, you know, whatever she had in her hand’ (Mae).

Here, Mae describes the act of RA as “evil”, however indicates her decision was made in the absence of sufficient information and guidance. For three participants, motivation to engage in RA was also related to attempt to secure their child's future stability and success. Grace describes how they kept their daughter in education:

we were driving her round about eight miles and, you know, holding down a full time job as well so there were lots of accommodation or whatever there that we wouldn't have had to do but, you know, she was in education…

Grace's rhetorical question “it's what you do isn't it? The role of a parent is not… that's what you do, you do support.” highlights her belief that this is their duty as a parent, perhaps challenging any narratives that suggest otherwise.

Participants often experienced the *global impact of relational accommodation on one's life*. They felt their whole existence and self‐concept are shaped by the persistence of BDD and supporting their child. This is experienced as a constant sense of uncertainty, inability to engage with other activities, and structuring their whole day around supporting their child:

Well, I can't do anything else… if I wanted to go to the gym, I have to think about how it fits in with‐ because obviously I work as well, I'm the breadwinner. How it fits in with work and picking [BDD sufferer] up at five thirty […] So, I'm taking out two hours of my day, every day. Two hours of my day is already accounted for by the two routines that I have… (Mary).

Mary demonstrates prioritising RA over her own leisure activities as a form of supportive parenting. Like Mary, others articulate the experience of “always” being ready to meet the demands of the BDD: I couldn't really concentrate a lot of the time because I was thinking about, you know, what was gonna happen next […] whether I was gonna have to go and pick her up (Robyn) and the restrictive nature of this on their own lives although you arranged to go somewhere, you always know there's the possibility it's gonna change, and it's gonna cancel. (Susan).

The experience of living with a child with BDD is scary, difficult and isolating, which left all participants *questioning one's experiences and identity*. For many, this involved doubting or questioning themselves in some way:

Five participants also described how having a child with BDD is marked by feelings of blame and that this meant they had “failed” as a parent:

I think… it's, it's made me, erm… question, question everything that I'd ever assumed or known. It's had a very profound effect …it's sort of questioned who I am, questioned my character, my morals, you know, I do feel responsible. (Kate).

RA is marked by the experience of guilt which led carers to “unravel the role of a parent” (Amelia) to understand BDD and how to play an active role in their child's recovery. This included accessing as much information as possible, ruminating on their experiences or talking it through with others: “I used to scroll the internet like comparing the mum's nets ‘does your daughter do this, does your daughter do that?’. You know, ‘is she looking in the mirror fifty thousand times a day?’ and things like that, you know?” (Susan). One participant who was “struggling” described engaging in the current research study as an attempt “to figure out what's going on” (Bailey). The alternative narrative was that participants' unique experiences have led to a positive shift in their identity and how they now respond to others: “It's changed me as a person. It has absolutely changed me and, and actually I'd say for the better. So I'm far more empathetic about, er, people's challenges” (Mary). Here, their experiences of caring for a child with BDD has been positively built into their self‐concept in a way that allows them to cope with the complexity and distress.

Participants' sense‐making around their experiences of RA and identity led to awareness of how they were *diverging from age‐related expectations* for both carer and loved one. Seven participants described how their experiences of RA disrupted age‐related expectations they hold for themselves and their child, which in turn were shaped by societal norms about increased independence:

It's just really, kind of, devastating because she does say, you know this, you know, umm, ‘I want to be doing things that a normal twenty‐four year old would be doing’. Who knows what those things are (laughs). But there is a, a perception of what you should be doing when you're a young person I suppose, and, and whatever we think that is in society… (Amelia).

This inevitably led participants to feel confronted with their own aging and mortality in the context of their children's current level of dependence on them. As Bailey notes, “I think now more than ever I'm so worried about my son being able to support himself (starts tearing up) when we're dead” but trying to support him to be more independent but balancing this with making sure that he doesn't lose his employment. Here, Bailey tries to determine how much of her input is helpful or detrimental to her son's growth and views RA as offering some quality of life (i.e., having a job). However, this can often be at the expense of carers own lives and independence: “So the fact that [Grace's daughter] hasn't gone to university, that she's lost at least two, maybe three years of education, erm, that's affected our life. You know, we'd have been in a different place. I'd have probably retired by now. Blah blah blah blah blah…” (Grace).

A substantial part of caring for a loved one with BDD (and perhaps heightened when caring for an adult) is *experiencing disempowerment as a carer*. Participants experienced their role as a mother suddenly being stripped away by medical professionals: “they [medical professionals] want to cut parents and carers out and that goes across all ages, er, but particularly when you're, you know, the person you're caring for turns eighteen” (Kate). They describe the difficulty they have accepting Western societal views that “someone turns eighteen and all of a sudden they can do all this stuff on their own, and the parents shouldn't be a part of it”, and that there needs to be a gradual stepping down of parental involvement to benefit the individual as opposed to being a “controlling helicopter parent” (Bailey). This extends to experiencing disempowerment in relation to clinical guidance not to accommodate BDD; describing an intellectual understanding of the guidance “I want to be the best parent I can. So, so when, when they say don't accommodate, I want to, to do that” but in reality, experiencing it as “overly simplistic and overly critical of parents” (Grace). Another likened her RA experience to a “battle” between her role as a parent and the BDD, which she equates to a “gremlin, which kind of sits on her [BDD sufferer] shoulder”:

The gremlin is… relentless. It's like a bottomless pit. […] I could be getting up at one in the morning, I could be running around the park, I could be driving to [city] and back. I could be, you know, going backwards and forwards to all the shops, buying the makeup or, you know, going to the get the stuff that's needed, but it will never be enough. It will never be enough for it. (Amelia).

In her lived world of constant conflict, it may have felt important to view the BDD as separate to her daughter to comprehend and cope better with the global demands it makes on her.

### Group experiential theme: ‘Relational gains and losses’

Participants described a distressing and traumatic environment in relation to the demands of being a mother supporting their child with BDD. The distress associated with BDD and RA permeates their family and wider systems. This inevitably draws them closer to those with whom they align with their experiences and needs but can also create tension and put strain on relationships. All participants described experiencing a *lack of compassion and understanding from others* in the context of their caring role, ranging from partners, relatives and friends to healthcare services. Kate described having to teach her friends about BDD, when what she requires from them is understanding, compassion and support. She developed an intolerance towards a lack of understanding and fear of judgement from others, which led her to “cut people out”. This is similarly endorsed by Mae; however, her frustration is around the gap between an intellectual understanding of BDD and lived experience: “even though you explain it to them, they don't really understand what it's like to live with someone that's got it unless they come and live with you for a while”. Mae also shared the polarising impact caring for her daughter with BDD had on her marriage. She felt that her partner “didn't have the understanding” of BDD and they had differing opinions around how to respond to BDD. Six participants experienced services as unhelpful and non‐collaborative in the context of their child being over 18: “the conversation didn't involve us sufficiently. Umm, you know, it wasn't, it wasn't very well managed” (Grace), even when participants had familiarity with healthcare systems: “I know what NICE guidance is. I know and, and I could not, I could not get what I needed to get…” (Robyn).

Furthermore, seven participants also spoke about the *impact on and from family and wider systems* (such as work and educational institutions) influencing how they respond to BDD. Parents described experiencing difficulties balancing responsibility and attention given to their child with BDD and their other children: “if I was showing more attention to [BDD sufferer] and her behavior, the other one [sibling] would say, ‘well, you know, you're always doing this for her, you're always doing that for her. You don't do that for me’” (Susan). Mae describes how her other children were pulled into RA at times: “I'd even resorted to leaving my youngest basically watching [BDD sufferer] to make sure that she was safe… there was no other way to, to get like grocery shopping in for example.” She was in a position of having to determine whether risk to her youngest child was less than her daughter with BDD, demonstrating her role as a protector and a provider and the pressure to meet all her children's needs. Similarly, Kate describes how different members of the family system would work together to avoid possible meltdown: “… if we're going out for lunch, then myself and my other daughter would walk, get the lunch, bring it back to the car, then we'd drive somewhere else so she could eat in the car”. Alternatively, some participants had positive experiences of wider systems accommodating BDD, feeling that people are on their side: “I mean, school made lots of modifications to try and help [BDD sufferer] attend the school and I think, I think the positive effect for [BDD sufferer] as a package is that she got her A levels” (Robyn), which may alleviate any feelings of failure as a parent.

Despite the felt lack of collaboration, compassion and understanding all participants shared, seven participants reflected on more positive experiences they had around the *specialness of community and shared experience*. Some participants reflected on their experience of joining a BDD carer support group and the impact it had in shedding the shame, stigma and isolation they experienced. This sense of camaraderie was particularly valuable for Susan who is navigating the situation as a single parent: “I felt there were other people out there… people were going, ‘oh, my son this’ or ‘my daughter that’ or, or ‘my sister this’ and so I found it helpful”. Kate described how much importance she places on actively seeking out connection with others who have a shared experience, to gain other perspectives, reduce isolation and avoid internalising a sense of blame or bad parenting: “Umm… So bearing witness for, erm, parents, sufferers, carers… hugely important… erm, without that, you internalise every single thing”. Alternatively, some participants described positive instances of sharing their experience with others who do not support a loved one with BDD: “I'm very lucky. I have really supportive friends who are really good and they live nearby… I think that's the thing that kind of gets me through a lot of the time.” (Amelia). Mae described using her acquired personal knowledge and experiences positively within the BDD community, which in turn helped shed the shame, isolation and avoidance of talking to others that she experienced for many years: “I enjoy helping people that are struggling or, you know… I know I'm not a clinician, but sometimes some parents just want to know there's someone else out there that is going through the same thing and I didn't have that”.

### Group experiential theme 4: ‘Understanding and experience of relational accommodation and BDD changes over time’

As described above, the experience of RA and caring for adult children with BDD is shaped by one's self‐concept, the joint distress experienced and the effect on relationships. Five participants relayed how one's understanding and experience of RA and BDD evolves with the passage of time, which can have implications on self‐concept, distress experienced and relationships with others. Participants described a need to adapt and be flexible to always changing needs: “… it's [RA behaviours] kind of changed over time. Erm, you know, I think different things came up at different times” (Amelia). Mary articulated that her experience of RA has reduced as BDD symptoms “naturally” decreased over time, therefore she has felt less distressed and more able to share her experiences: “had we been having this conversation five years ago, erm… I, I would have been in a different place”. Other participants reflected on how their *understanding* of RA and BDD has developed over time. Grace observes that she “used to think that mental illness there was, you know, it was a failure of parenting basically” but that she is no longer so judgmental and recognises that it is “more complicated than that.” (Grace). Grace's understanding of mental illness as a failure of parenting likely significantly impacted her self‐concept and sense‐making around her own experiences. Grace suggests accepting BDD into their lives has been a process, eventually experienced as a shift in blame. Interestingly, participants demonstrated an evolved understanding and awareness of RA, “with hindsight, erm, and knowing a lot more about BDD, erm, I would say none of it [RA] was helpful.” (Mae), an experience shared by other participants, “did that make it worse, carry on?… I don't know, I still look back and think, could we have done things differently?” (Robyn). Robyn concludes by reflecting on her limited capacity within a highly stressful context, “I'm not sure that we could have done with everything else that was going on”.

## DISCUSSION

This qualitative study is the first to explore the impact of adult BDD and RA on caregivers, and specifically: (1) The experiences of family members and SO living with an adult with BDD/appearance‐related anxiety; and (2) How family members and SO respond to the symptoms of BDD/appearance‐related anxiety in their relative/loved one. Results suggest the experience of mothers caring for their loved one with BDD is distressing, disempowering and isolating. They face tension between wanting to care in ways that may be labelled RA, with clinical guidance labelling this care as harmful, *and* between using RA to reduce their child's distress, which is debilitating to their own lives. Over time, experience and understanding of RA may evolve, offering new perspectives. Connecting with others who are understanding and compassionate can reduce the burden of RA on caregivers. Due to limited BDD literature and diagnostic overlap with OCD, findings are discussed primarily in relation to OCD.

Most participants described the challenges of living and caring for someone with BDD as long‐lasting, isolating and disempowering. All attempted to make sense of their experiences and parental role, often leading to self‐blame and feelings of parental failure and powerless in their child's recovery. This aligns with paediatric BDD literature, highlighting the negative impact RA has on parents' well‐being, including desperation, helplessness and powerlessness (Jassi et al., 2020). However, two participants described positive personal changes, such as gaining a unique perspective and greater empathy towards others. Some transformed their challenges into positives within the BDD community, such as advocacy work, spreading awareness via media outlets and charity work supporting other caregivers. Previous literature failed to capture these nuances in the caregiver experience, which potentially highlights the importance of supporting parents to access such opportunities to protect their wellbeing.

The results suggested all parents engaged in RA of their child's BDD symptoms, consistent with adult OCD literature (Lebowitz et al., 2016). Some participants reduced or stopped RA as their child's BDD symptoms decreased or remitted. It is interesting to consider literature exploring the relationship between RA and OCD symptom severity in this context. Wu et al. (2016) suggest a bi‐directional relationship; however, this study found parents reduced RA only *after* their child's BDD symptoms and/or risk decreased. Another study investigating maternal accommodation in adolescents with BDD found only a small correlation with BDD symptom severity, and no evidence of an association with treatment outcomes (Hogg et al., 2024), raising questions about whether reducing RA plays as significant a role in BDD treatment as OCD treatment.

All participants articulated awareness of clinical guidance around reducing RA and its rationale but felt ambivalent about following it. Several themes emerged that may explain this. All participants described an intertwined relationship between their self‐concept and response to BDD, believing that RA reflects compassion, support and good parenting, particularly in the context of risk, prioritising their child's safety above clinical guidance. Five participants shared traumatic experiences related to their child. Symptom improvement made them more fearful of relapse or re‐experiencing traumatic scenarios. This led participants to engage in excessive supervision of their loved one and hypervigilance around symptom increase. This aligns with paediatric BDD literature describing risk management and suicidality as key factors in RA of BDD (Jassi et al., 2020). This is likely to be more specific to BDD given the higher suicidality risks associated with it (Phillips, 2007; Phillips & Menard, 2006). Five participants described heightened distress influencing their response to BDD. Increased RA of OCD symptoms and low parental distress tolerance are associated (Caporino et al., 2012; Kagan et al., 2017), which may similarly occur in parents of adults with BDD. Without adequate support or guidance, parents accommodated BDD symptoms to try and minimise their child's and their own distress, including guilt, blame and helplessness. Distress tolerance is typically understood as managing one's own emotions; however, parents must also manage their child's distress, especially in younger children with limited self‐regulation (Morris et al., 2007). Given the study's findings that adults with BDD experience reduced independence due to debilitating anxiety, carers may use RA to manage distress. Five participants conveyed how their understanding and experience of RA had evolved over time. This was partly due to changing symptoms and unpredictability around the daily support needed, but also how their understanding of RA and BDD developed. For some, this involved a shift in self‐blame, whilst others questioned the helpfulness of RA and felt more empowered to resist accommodating BDD. This emphasises the importance of psychoeducation for SO around RA and BDD at the beginning and throughout treatment, to empower and increase individuals' agency for change.

Most participants were aware of the discrepancy between developmental age and life stage. Participants often compared their child to their peers in terms of ability and independence. This is likely to be specific to caregivers of adults with BDD compared to the paediatric literature, given the dependence children and adolescents inherently have on their parents. This response may also be shaped by upbringing in an ableist society, impacting social reactions to disability (Shakespeare, 2006). Similarly, participants compared themselves to peers, noting reduced independence and lifestyle disruptions due to caregiving. Similar themes were found in the paediatric literature on the impact of RA on finances, work and leisure activities (Jassi et al., 2020). Participant demographics of this study may have moderated this finding. Motivations for engaging in RA behaviours that supported their child to maintain education, employment and independence may reflect sociocultural beliefs about good parenting and socialisation goals. Research shows parents in urban, industrialised societies value independence, whereas those in rural societies value interdependence (Keller et al., 2006). These study findings may be specific to White Western mothers of an adult with BDD. It is important to amplify the voices of less privileged caregivers to understand their experiences of RA.

Participants described the pervasive nature of RA of BDD symptoms. Brownings et al. (2023) highlight the “controlling nature of OCD” over family functioning, whilst Futh et al. (2012) found parents likened OCD to addiction or obsession. This persistence, alongside the distress of managing severe BDD symptoms, permeates their entire family and wider systems. Participants noted how these challenges both strengthened and weakened relationships. Many described negative consequences on the whole family system, with siblings and partners feeling marginalised due to caregivers' focusing on the individual with BDD. These experiences align with reduced sibling attention (Brownings et al., 2023) and parental marital issues (Futh et al., 2012) within paediatric OCD literature, and increased conflict and sibling frustration within paediatric BDD literature (Jassi et al., 2020). This highlights the need to explore the impact of RA beyond the parent–child dynamic.

All participants described accounts of isolation, invalidation and frustration in their caring role, especially towards partners, friends and, importantly, healthcare professionals, affecting their ability to provide optimal care. Conversely, they valued connecting with others through their unique and shared lived experience, such as through support groups or simply talking to others who demonstrate non‐judgement and compassion towards the overwhelming and confusing nature of their caring experience and engagement in RA. These relational experiences align with OCD literature around a lack of shared familial, professional and societal understanding and the importance of talking to understanding people (Sowden et al., 2023). Peer support, well‐recognised in specialist BDD services and charities, is based on the idea that individuals sharing similar experiences can connect, support and learn from one another (Gillard, 2019). This offers valuable insights into improving existing treatment, focusing on compassionate care for caregivers/SO of individuals with BDD. Relatedly, involving loved ones in the development and implementation of RA‐focused interventions may help ensure that their perspectives are acknowledged and validated. A co‐production approach may increase the acceptability and effectiveness of such interventions, by ensuring that strategies to reduce RA are sensitive to the interpersonal challenges faced by loved ones and are feasible within real‐world relational contexts. A key finding of this study specific to the adult BDD population was the disempowerment carers experienced. Carers of adults may face unique challenges; for instance, adults are generally more able to present an emotionally charged, intellectual argument around why parents should accommodate and are potentially able to physically intimidate their parent depending on aspects of their identities, whereas children may be less able to engage in such interactions. Adult caregivers may be excluded from healthcare services where their child is over 18, or have their concerns dismissed when their child minimises symptoms due to poor insight (Phillips et al., 2012). This suggests a missed opportunity to improve the transition between paediatric and adult services; however, it is important to recognise structural factors influencing this, such as service constraints affecting families *and* clinicians. Parents also felt clinical guidance around RA undermined their maternal instincts, finding it overly simplistic and critical. Similar issues are seen in OCD literature, where parents felt reprimanded or blamed by professionals, and professionals felt limited in their capacity supporting parents with reducing RA (Sowden et al., 2023). These findings highlight the emotional and moral complexity of maternal caregiving overlooked in clinical RA guidance and suggest a need for clinical approaches that move beyond simply reducing RA, instead offering alternatives that maintain emotional connection without reinforcing BDD‐related processes. Drawing on OCD literature, this may involve emphasising forms of emotional support that validate distress without engaging in reassurance (Halldorsson & Salkovskis, 2023), which may help align RA reduction with loved ones' desire to respond in compassionate and supportive ways.

Although this research aimed to explore the experiences of diverse family members and SO living with an individual with BDD (such as partners, siblings and friends), the sample consisted solely of mothers. This may be due to societal norms positioning mothers as primary caregivers, making them more likely to share their experiences. All mothers reported lack of collaboration, compassion and understanding from others and many spoke about shouldering all responsibility, even with another parent present. This may have motivated their participation to raise awareness and have their voices heard. Although a small, homogeneous sample with relevant lived experience who can articulate their experience is appropriate for an IPA study, the limited diversity may mean that issues of marginalisation and social exclusion were less discussed, limiting the transferability. In particular, as participants were recruited via a BDD support group, the experiences of the most marginalised and isolated carers may not be well represented. Future research should examine different relationships to the individual with BDD (beyond the parent–child dynamic) and the intersection of this with gender, ethnicity and socio‐economic status would go some way to address these limitations. Similarly, given that experiences of RA change over time, utilising a longitudinal approach could provide richer accounts of change and enable identification of factors which promote positive trajectories towards enhanced quality of life (Calman et al., 2013), especially as the individual with BDD progresses through treatment or their condition changes. This could help inform targeted interventions for different caregiving stages. Further, future research should explore how alternative support strategies, such as those derived from OCD literature on reassurance, can be adapted and evaluated in the context of BDD. Future research should continue to explore the emotional toll of caregiving, including guilt, isolation and burnout to guide support systems and interventions for both practical and emotional caregiver needs. Including carers in the research process would ensure this is relevant and grounded in real‐world experiences.

Although preliminary, the study highlights the significant impact of BDD on mothers, suggesting the need for parent‐specific support. Parents often viewed RA as good parenting by alleviating their child's distress yet struggled to differentiate between caregiving and accommodating BDD symptoms. RA was often understandably a function of reducing their child's risk. Support should include practical advice for managing BDD symptoms without enabling harmful behaviours, such as training on setting boundaries, managing distress and fostering positive coping mechanisms. Currently in services, carers may be invited to join psychoeducational sessions learning about BDD or asked to take a co‐therapist position in their child's care but often lack resources or guidance around what to do. Screening for RA behaviours and including this in psychological formulations may help carers better understand how they may be drawn into BDD and how to target this in treatment. Close risk management while undertaking this work is also essential. A focus on self‐care and mental health resilience is crucial to empower caregivers to manage their own needs. Coping strategies like externalising BDD from their loved one may help reduce emotional burden and improve communication and relationship dynamics (Roth & Epston, 1996). Compassion, sensitivity and non‐judgement somewhat alleviated carers' distress, highlighting the need for parent‐focused interventions integrating approaches such as compassion‐focused therapy (CFT) to support caregivers, with the aim of reducing self‐blame and guilt (Shenaar‐Golan et al., 2021). Carer support groups are vital, ensuring they are external to mental health services so carers can benefit as early as possible and accessible for those facing financial or geographical barriers. These groups can reduce isolation, promote shared learning and help caregivers navigate the emotional toll of caregiving.

## CONCLUSION

This exploratory study offers preliminary insights into how a small group of mothers experience RA in relation to their adult children with BDD. Findings indicate that RA is linked to self‐concept, distress avoidance and risk concerns, which aligns with the paediatric BDD and/or OCD literature. Parental exclusion from healthcare services and age‐related expectations are likely to be specific to adult caregiver populations. Collectively this is experienced as disempowering and isolating, affecting the entire family and wider systems. Accessing a compassionate and understanding community may help buffer these effects. Novel findings tentatively suggest that RA experiences evolve over time, highlighting the need for ongoing assessment and support for RA throughout treatment. This research emphasises the need for improved BDD treatment protocols, more nuanced clinical guidance around RA and better support for caregivers. Policymakers should develop clear, accessible guidelines for healthcare professionals to support carers more effectively with tailored interventions, addressing the unique challenges they face.

## AUTHOR CONTRIBUTIONS

**Deanna Fallah:** Conceptualization; methodology; formal analysis; data curation; investigation; writing – original draft; writing – review and editing; project administration. **Lucy Hale:** Conceptualization; methodology; validation; writing – review and editing; supervision. **Francesca Cicconi:** Writing – review and editing; supervision. **Hannah Frith:** Conceptualization; methodology; validation; writing – review and editing; supervision.

## CONFLICT OF INTEREST STATEMENT

The authors declare no conflicts of interest.

## ETHICS STATEMENT

Ethics approval was granted from the University of Surrey's Research Integrity and Governance Office (RIGO) (EGA ref.: FHMS 22‐23236 EGA) prior to data collection.

## Supporting information


File S1.

## Data Availability

The research data is unavailable to access as it includes sensitive or confidential information.
