# Supplementary material for: Understanding the experience of relational accommodation for caregivers of an individual with body dysmorphic disorder: An interpretative phenomenological analysis
Source: Psychol Psychother. 2026 Apr 23;99(3):1050–67. doi: 10.1111/papt.70067 (PMC13456364; doi:10.1111/papt.70067)
Supplement: Supplementary file 1 [file PAPT-99-1050-s001.docx]

**Supplementary Materials**

Supplementary File 1

*Interview topic guide*

**Relational Accommodation in Body Dysmorphic Disorder**

Thank you for agreeing to take part in this research project. We are trying to find out about what life is like for caregivers/significant others of a person with Body Dysmorphic Disorder (BDD). We appreciate this can be hard for everyone in the support network, including you, but we are keen to understand your experience to increase our knowledge about how family members/significant others experience and respond to their loved one’s BDD. This information may help us improve the way BDD treatment is delivered to adults with BDD and families in the future.

1. Before we talk about your personal experience of caring for someone with BDD, what is your general understanding of BDD? How long have you been caring for someone with BDD?

2. Are there specific ways you adapt your behaviour in response to your loved ones BDD? If so, what sort of things do you do or avoid doing?

Prompt:

- Does your loved one ask you to do things that it is hard for them to do?
- What do you do when your loved one wants to avoid things which make them anxious?
- Do you avoid talking about certain things so you don’t trigger your loved one’s thoughts about their appearance or behaviours?
- Do you provide items / things for your loved ones BDD behaviours?
- What motivates you to respond in these ways? Do you think these responses have any positive or negative impact on your loved one?

3. How does doing these things make you feel?

*Prompt:* *check for each example mentioned in Q5*

4. Has doing these things affected your day-to-day life in any negative or positive ways?

*Prompt: explore the positive and negative examples given.*

*Prompt: have you had to stop or reduce doing the things you enjoy because of your loved one’s BDD?*

5. Has doing these things impacted on you as a person?
6. Has anything been helpful to support you in your caring role?

What advice would you give to somebody who is caring for someone with BDD?
